# Antimicrobial, Cytotoxic, and Antioxidant Potential of a Novel Flavone “6,7,4′-Trimethyl Flavone” Isolated from *Wulfenia amherstiana*

**DOI:** 10.1155/2020/3903682

**Published:** 2020-03-08

**Authors:** Maria Kakar, Muhammad Usman Amin, Saad Alghamdi, Muhammad Umar Khayam Sahibzada, Nisar Ahmad, Naseem Ullah

**Affiliations:** ^1^Department of Pharmacy, Abasyn University, Peshawar, KPK 25000, Pakistan; ^2^Laboratory Medicine Department, Faculty of Applied Medical Sciences, Umm Al-Qura University, P.O. Box 715, Makkah 21955, Saudi Arabia; ^3^Department of Pharmacy, Sarhad University of Information Technology, Peshawar, KPK 25000, Pakistan; ^4^Islam College of Pharmacy, Sialkot, Punjab, Pakistan

## Abstract

*Wulfenia amherstiana* belongs to the Scrophulariaceae family and various plants of this family are known for their biological activities. The present study was focused on the isolation of bioactive compounds including a novel flavone 6,7,4′-trimethyl flavone (TMF) along with three known flavonoids such as quercetin, rutin, and a steroid *β*-sitosterol which were isolated from the ethanolic extract of *W. amherstiana* (Himalayan Wulfenia) through column chromatography and purified by using HPLC. Their structures were identified and elucidated through electron ionization mass spectroscopy (EIMS), 1DNMR (^1^H-NMR and ^13^C-NMR), and 2DNMR (COSY, HMQC, and HMBC) spectroscopy. The antimicrobial activities of this novel compound were evaluated through agar well diffusion method, while antioxidant and cytotoxic activities were assessed through 2,2-diphenyl-1-picrylhydrazyl (DPPH) free-radical scavenging assay and brine shrimp lethality assay, respectively. The NMR data revealed that TMF is a novel compound. TMF showed potential antibacterial and antifungal activities against *Staphylococcus aureus* (MIC = 128 *μ*g/ml) and *Candida albicans* (MIC = 128 *μ*g/ml). The cytotoxic potential of TMF was determined from brine shrimp lethality assay with LD_50_ of 127.01 *μ*g/ml. The free-radical scavenging potential of TMF at various concentrations implicated its strong antioxidant activity in vitro. The results revealed that TMF demonstrated substantial antimicrobial activity against *S. aureus* and *C. albicans*, strong antioxidant activity, and moderately cytotoxic activity.

## 1. Introduction

There are many compounds available in the current drug market for the treatment of different diseases ranging from minor to severe. The metabolites, either secondary or primary, isolated from plants are also used as medicines [1]. Even these compounds are used widely in all pharmaceutical products. A study in USA has revealed that 2/5^th^ of medicines are composed of these plant secondary metabolites such as flavonoids, terpenoids, sterols, phenols, and alkaloids [2].

The Scrophulariaceae family is composed of various flowering plants. This family is found all around the world, having 275 genera and 5,000 species [3]. Scrophulariaceae is known for its medicinal uses. The medicinal importance of the family is because of various biologically active compounds such as terpenoids, flavonoids, steroids, phenylethanoids, iridoid glycosides, saponins, and minerals. The presence of such a diverse range of compounds can be attributed to several biological activities like anti-inflammatory, anticancer, antioxidant, antimicrobial, antiviral, and antihyperlipidemic activities [4].

*W. amherstiana* belongs to the Scrophulariaceae family [5] and in Pakistan, it is found in Swat, Hazara, Murree, Poonch, and Kashmir. This plant was named after the botanist Franz Xaver von Wulfen who discovered it. The literature reveals no data regarding this plant's biological activities, but other species of this family like *W. carinthiaca* have been reported to have antinociceptive, anti-inflammatory, and antioxidant effect due to various glycosides present in it [6]. *Anarrhinum orientale* is another plant of the same family; various iridoid glycosides were isolated from its methanolic extract. These compounds were found to inhibit hepatitis C (HVC) protease activity [7]. The present study was aimed at the evaluation of biological activities of a new compound, i.e., 6,7,4′-trimethyl flavone (TMF), isolated from the plant *W. amherstiana*.

## 2. Materials and Methods

### 2.1. Chemicals

Standard bacterial strains used are *Staphylococcus aureus* NCTC 6571 (*S. aureus*), *Streptococcus pneumoniae* NCTC 7466 (*S. pneumoniae*), *Bacillus subtilis* (*B. subtilis*) NCTC 8236, *Pseudomonas aeruginosa* (*P. aeruginosa*) ATCC 10145, *Salmonella typhi* (*S. typhi*) ATCC 6539; fungal strains used were *Trichophyton longifusis* (*T. longifusis*) clinical isolate, *Candida albicans* (*C. albicans* ATCC 2091), *Aspergillus flavus* (*A. flavus* ATCC 32611), *Microsporum canis* (*M*. *canis* ATCC 11622), *Fusarium solani* (*F. solani* ATCC 11712), and *Candida glabrata* (*C. glabrata* ATCC 60406). The Pakistan Council of Scientific & Industrial Research (PCSIR) laboratories, Peshawar, KPK, Pakistan, provided all these standard strains.

Media used were Sabouraud dextrose agar (SDA), nutrient agar (NA), and nutrient broth (NB). Standard antibiotics cefazolin and kanamycin and standard antifungals amphotericin-B and miconazole were bought from Sigma-Aldrich. Different solvents chloroform, ethyl acetate, *n*-butanol, *n*-hexane, and dimethyl sulfoxide (DMSO) were purchased from Merck (Germany).

### 2.2. General Experimental Procedure

#### 2.2.1. Physical Constants

Melting points (MP) were measured by MP apparatus (Buchi 535).

#### 2.2.2. Spectroscopy

The nuclear magnetic resonance (NMR) experiments were performed on NMR equipment (Bruker AMS, 400 MHz) using both 1DNMR (^1^H-NMR and ^13^C-NMR) and 2DNMR techniques (COSY, HMQC, and HMBC). The chemical shifts were measured in PPM with tetramethylsilane (TMS) (standard) and measurement of scalar-coupling constants (*J*) was in hertz (Hz). Various solvents such as deuterated chloroform (CDCI_3_), deuterated methanol (CD_3_OD), and pyridine-d5 were used in the Nuclear Magnetic Resonance (NMR) spectroscopy. Electron ionization mass spectroscopy (EIMS) was conducted on a Jeol JMS HX 1 l0 mass spectrophotometer.

#### 2.2.3. Chromatography

Column chromatography was performed on silica gel-60 (70–230 Mesh, by Merck). Thin-layer chromatography (TLC) was done on precoated Kieselgel-60 GF_254_ aluminum (Al) sheets made by Merck. The ceric sulphate (CeSO_4_) solution was sprayed for the visualization of spots and then subjected to heating, while a high-performance liquid chromatography (HPLC) device (Shimadzu SHP-600), coupled to a photodiode array detector 20-A, was employed for confirming the sample's purity of all the isolated compounds. The HPLC analysis was carried out under isocratic conditions using a C8 column (4.6mm × 150mm) packed with 5 *μ*m diameter particles; the mobile phase was acetonitrile : water (95 : 5, v/v) containing 1.0% acetic acid with the flow rate = 1 ml/min and the run time was set for 50 minutes. Detections were made at 365 nm [8].

### 2.3. Collection and Identification of the Plant

The *W. amherstiana* (whole plant) was collected from Bara Gali in the northern areas of Pakistan in August. The plant taxonomist Dr. Muhammad Ibrar identified the plant at the Department of Botany, Peshawar University, KPK, Pakistan. The plant was identified by its purple flowers that were tubular in shape with spikes on one side; the leaves were oblong in shape, crowded at the base. A specimen (Voucher no. WA450987) of the plant was also placed at the herbarium of the Department of Botany in the same university.

### 2.4. Pretreatment of the Plant's Materials

The whole plant including flower was dried under shade in fresh air for several days and then chopped into small pieces. The small pieces were pulverized to ﬁne powder and soaked in ethanol for several days. About 5 kg of the plant material was taken and extracted with 2600 ml ethanol. Using a rotary evaporator, it was concentrated to a semisolid mass weighing 175 g. About 80 g of it was loaded on large column packed with silica gel (stationary phase). The elution of column was done with 100% *n*-hexane obtaining fractions 1–73. Each fraction was tested for the presence of compounds using TLC plates with different solvent systems. Initially, 1–73 fractions did not show any compound in it. Furthermore, the same column was subjected to elution by enhancing the polarity of the solvent system using *n*-hexane/chloroform. From this, 74–95 fractions were obtained which were analyzed for the presence of compounds, i.e., compounds 1 and 2. The polar fractions of chloroform : methanol (50 : 50) was visualized on a TLC plate using *n*-butanol : acetic acid : water (BAW) (12 : 3 : 7) as a solvent system. This showed only one component, while other components present in the fraction showed a black streak on TLC before and after visualization with ceric sulphate, UV light, and daylight. The yellow-colored material was dissolved in methanol : water (9 : 1), washed repeatedly, and precipitated. The compound was dissolved in methanol : water (9 : 1) and loaded on a TLC plate and developed in the methanol : water : chloroform (9 :1 : 5) system. The visual streak was scratched from the glass plate and again subjected to preparative TLC using the BAW system. The desired compound thus isolated showed *R*_*f*_ value = 0.72 by cospotting technique. Fractions obtained from the main column by eluting with chloroform : methanol (70 : 30) seemed less resolved and therefore were combined together and again subjected to further chromatography using chloroform : methanol (9 : 1). This attempt resulted into seventy fractions, but the last 20 fractions (74–95) contained the compound. However, due to indecisive profile of TLC result, it was further chromatographed until this exercise ended in partially pure fraction. This fraction was concentrated and additionally purified through preparative TLC (in chloroform : methanol, 8 : 2), and yellow-colored spots were isolated in the form of amorphous powder after concentration in vacuum. An *R*_*f*_ value of 0.6 was calculated by cospotting with the reference sample.

Moreover, elution with chloroform : methanol offered fractions 96–116 which gave compounds 3 and 4. The compound 3 was isolated from fractions 96–116 from the main column. These fractions were combined and subjected to column chromatography (chloroform : hexane, 8 : 2). The yellow spot contained closely related two more spots having *R*_*f*_ values of 0.62 and 0.52, respectively. They were subjected to preparative TLC (chloroform : methanol, 3 : 4). The yellow-colored compound was scratched from TLC and isolated by following the routine procedure. These fractions obtained from n-hexane : chloroform (2 : 8) showed a yellow spot of relative greater density. The similar fractions were combined and subjected to column chromatography (chloroform : n-hexane, 8 : 2). The yellow color was composed of two spots having *R*_*f*_ values (0.62 and 0.52). They were subjected to preparative TLC (chloroform : methanol, 3 : 4). The yellow-colored compound was scratched from the TLC plate and purified.

Finally, elution with methanol was performed which was checked on TLC using *n*-butanol : acetic acid : H_2_O (BAW) in a ratio of (12 : 3 : 7) and weak spots were observed, showing no compounds.

### 2.5. Phytochemical Screening of Crude Plant Extract

The Liebermann–Burchard test was adopted for steroids [9], while Mayer's and Wagner's tests were performed for the detection of alkaloids [10]. The saponins were confirmed through the Frothing Test and RBC hemolysis [11, 12]. The flavonoid confirmation was carried out through NH_3_ and vanillin HCl tests [13, 14]. Salkovaski and Liebermann–Burchard tests were performed for detecting triterpenoids [15, 16].

### 2.6. Antibacterial Assays (Agar Well Diffusion Method)

The agar well diffusion method was adopted for testing the bacterial strains sensitivity against TMF. The compound was dissolved in ethanol at different concentrations and then poured into wells made by using a sterilized borer through a micropipette. The bacterial strains were inoculated in 10 ml NB in test tubes and subjected to incubation at 37°C for 24 hours. Bacterial lawns were prepared on NA plates [17]. The wells were filled with the specific concentrations (50 *μ*g/ml, 100 *μ*g/ml, 200 *μ*g/ml, 300 *μ*g/ml, 400 *μ*g/ml, 500 *μ*g/ml, and 600 *μ*g/ml) of the compound on the NA plates before incubation. After this step, the inhibitory zones were observed for measurement and properly documented. Only those concentrations of the test compound were considered at which the maximum zone of inhibition was observed. For positive control, cefazolin was used for Gram-positive bacteria and kanamycin for Gram-negative bacteria; the solvent was used as negative control [18].

### 2.7. Antifungal Assay (Agar Well Diffusion Method)

Antifungal activity of TMF was assessed against all the fungal strains under study. All fungal strains were inoculated on SDA. Through the sterilized borer, wells were made in these plates. TMF dissolved in ethanol was poured into each labeled well at specific concentrations (50 *μ*g/ml, 100 *μ*g/ml, 200 *μ*g/ml, 300 *μ*g/ml, 400 *μ*g/ml, 500 *μ*g/ml, and 600 *μ*g/ml). The plates were subjected to incubation at 35°C for 72 hours, and inhibitory zones were measured and recorded in triplicate to have average values. Ketoconazole at a concentration of 100 *μ*g/ml was used as positive control in the experiment and ethanol as negative control [19].

### 2.8. Minimum Inhibitory Concentration (MIC) Assay

MIC assay was carried out by the macrobroth dilution method. Different concentrations of TMF were used ranging from 8 *μ*g/ml to 1024 *μ*g/ml. Following the addition of test materials to sterile test tubes, bacterial and fungal cultures were prepared as mentioned earlier and were incubated overnight at 37°C. Absence of any visible growth in test tubes was considered as the MIC of test material [20].

#### 2.8.1. Cytotoxic Assay

The cytotoxic activity of the new compound (TMF) was assessed through brine shrimp lethality assay [21]. This assay is based on the number of brine shrimp larvae killed by the experimental compound. The brine shrimp eggs were hatched in sea water after incubation for 24–72 hours. Various concentrations (10 *μ*g/ml, 50 *μ*g/ml, 100 *μ*g/ml, and 1000 *μ*g/ml, respectively) of the compound were taken in separate clean test tubes. 30 brine shrimps were added to each test tube containing 5 ml sea water. As a positive control, etoposide, a cytotoxic agent was used and sea water was negative control. The number of shrimps found dead was counted after 24 hours, and percentage mortality was determined by the following formula [22].

LD_50_ was calculated through probit analysis:

(1) $$\begin{array}{c} \%\text{mortality}=\frac{\text{number of dead or immobile brine shrimps}}{\text{total number of brine shrimps}}\times 100. \end{array}$$

### 2.9. Antioxidant Activity

The antioxidant activity was performed through 2,2-diphenyl-1-picrylhydrazyl (DPPH) free-radical scavenging activity. Various concentrations of test compound were used and mixed with 0.5 ml of 1 *μ*M/L of 2,2-diphenyl-1-picrylhydrazyl (DPPH) solution in methanol. All the compound solutions were incubated at room temperature (in darkness) for 30 minutes and then subjected to analysis by using an ultraviolet spectrophotometer (Shimadzu UV-VIS 1200 spectrophotometer (Shimadzu Corp., Kyoto, Japan)) at 517 nm [23]. Ascorbic acid was used as standard. The scavenging activity was determined through the formula given as follows:

(2) $$\begin{array}{c} \text{effect of scavenging}\left(\text{\%}\right)=\frac{1-\text{ sample}\left(517\text{nm}\right)}{\text{ control}\left(517\text{nm}\right)}\times 100\text{.} \end{array}$$

## 3. Results and Discussion

### 3.1. Phytochemical Screening

The screening test confirmed the presence of different compounds as shown in Table 1. It was clearly evident that flavonoids, steroids, saponins, and triterpenoids were present in the plant ethanolic extract, while the test for alkaloids was negative.

### 3.2. Chromatography

#### 3.2.1. Column Chromatography of the Crude Ethanolic Extract of *W. amherstiana*

The dried crude ethanolic extract (80 g) was placed onto a silica-gel column, and elution was done with *n*-hexane for the extraction of nonpolar and oily components of the extract. Polarity was gradually increased using chloroform (5%, l0%, 25% up to 100%). Fractions obtained from n-hexane and chloroform were concentrated through a rotary evaporator and checked on TLC plates. The TLC plates were visually analyzed with ceric-sulphate and UV light for locating the spots.

#### 3.2.2. HPLC Analysis

**Figure 1:**
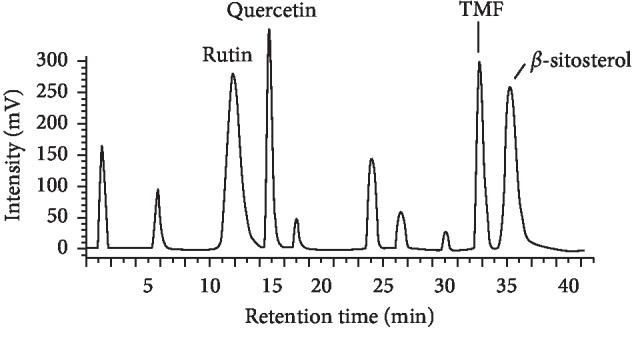
High-performance liquid chromatography (HPLC) profile of the ethanolic crude extract of *W. amherstiana*.

HPLC profile of the ethanolic extract was acquired with well-resolved peaks as shown in Figure 1. The compounds in the subfraction were identified as rutin (retention time (*t*_R_) = 12 min), quercetin (*t*_R_ = 15 min), 6,7,4′-trimethyl flavone (*t*_R_ = 33 min), and *β*-sitosterol (*t*_R_ = 36 min). Identification of compounds in the ethanolic crude extract chromatogram was made on the basis of retention time of individual pure compound isolated.

### 3.3. Identification of Isolated Compounds

#### 3.3.1. Structure Elucidation of Compound 1

**Figure 2:**
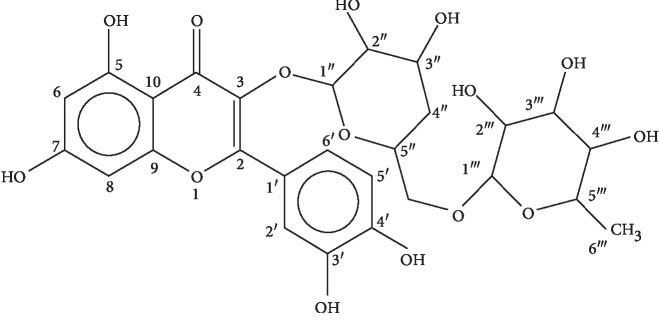
Chemical structure of rutin.

The mass spectra of compound 1 showed peaks at *m*/*z* 301, 179, and 151 conforming to molecular formula C_27_H_30_O_16._ Further confirmation was achieved by determining its MP (109–111°C) and analyzing it by spectroscopic methods. The ^1^H-NMR (400 MHz, CD_3_OD) and ^13^C-NMR (CD_3_OD, 100 MHz) data of compound 2 have been shown in Table 2. Compound 1 was identified as rutin as revealed in Figure 2. As rutin is a known compound, its spectroscopic data such as EIMS, ^1^H-NMR, and ^13^C-NMR were in compliance with the previous studies [24, 25].

#### 3.3.2. Structure Elucidation of Compound 2

**Figure 3:**
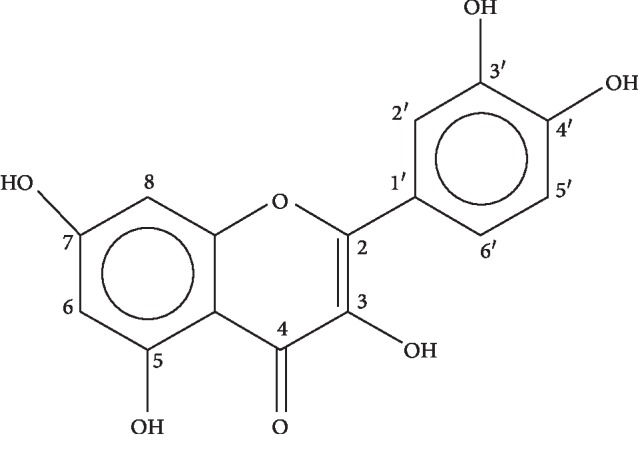
Chemical structure of quercetin.

The characteristic ions of compound 2 as revealed by MS were at *m*/*z* 301, 271, 255, and 179. The molecular formula was C_15_H_10_O_7_ (MP 316-317°C). The ^1^H-NMR (400 MHz, CD_3_OD) and ^13^C-NMR (CD_3_OD, 100 MHz) data of compound 2 have been shown in Table 3. The ^1^H and ^13^C-NMR data were in compliance with a study where ^1^H-NMR and ^13^C-NMR spectra of various flavonoids were obtained, which were isolated from *Agrimonia pilosa* Ledeb [25, 26]. Through the spectroscopy results, compound 2 was identified as quercetin (Figure 3).

#### 3.3.3. Structure Elucidation of Compound 3

The electron mass spectra of this compound had shown peaks at *m*/*z* 298, 276, 258, 254, and 149 corresponding to molecular formula C_18_H_12_O_3_ (MP 285–286°C). The ^1^H-NMR (400 MHz, CD_3_OD) data of the compound have been shown in Table 4. It revealed that there were 02 aromatic doublets at PPM 7.26 (d, H-2′) and 7.18 (d, H-5′), 03 methyl groups at PPM 1.20 (3H, s), 1.51 (3H, s), and 1.51 (3H, s), and 01 singlet hydroxyl group at PPM 12 (1H, s, 5OH). It has 04 singlets at PPM 6.71 (s, H-3), 6.60 (s, H-8), 7.18 (s, H-3′), and 7.26 (s, H-6′). The presence of hydroxyl group is a characteristic of flavone which is attached to the carbonyl group. The ^13^C-NMR (CD_3_OD, 100 MHz) data revealed the presence of 12 aromatic carbons along with the 03 methyl groups at PPM 11.0 (C-11), 19.4 (C-12), and 21.3 (C-7′), 06 methine carbons at PPM 104.5 (C-3), 106.5 (C-8), and 127.5 (C-2′), an unsaturated carbonyl carbon at PPM 182.1 (C-4), and 08 quaternary carbons at PPM 163.6 (C-2), 117.2 (C-6), 144.7 (C-7), 155.5 (C-9), 109.8 (C-10), 127.0 (C-1′), and 137.6 (C-4′).

**Figure 4:**
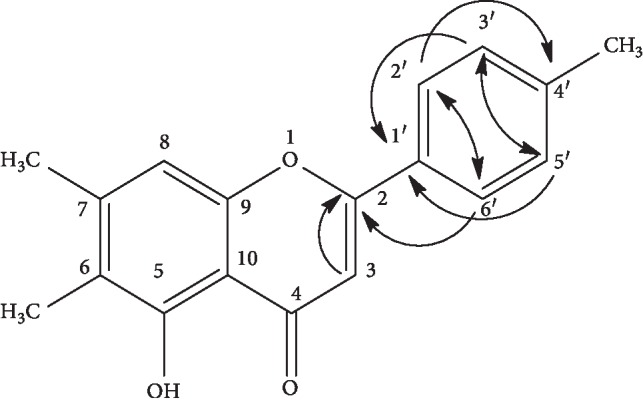
Chemical structure of 6,7,4′-trimethyl ﬂavone (TMF).

The spectral data of compound 3, as presented in Figure 4, revealed it as a new compound and was named 6,7,4′ tri-methyl ﬂavone (TMF) with no previously reported data regarding its structure and biological or pharmacological activities.

(2)

^1^H-NMR data of this new compound was further supported by 2D NMR techniques such as COSY, HMQC, and HMBC correlations. COSY revealed a correlation between H-2′ and H-6′ at PPM 7.26 and H-3′ and H-5′ at PPM 7.18 and produced a signal describing monosubstitution on the aromatic ring.

The HMQC has provided information about the correlations between protons and carbons of the novel flavone isolated from the plant under study. The proton present at C-3 is basically the characteristic of flavones, which was proved through correlation of proton at PPM 6.71 on H-3 with carbon C-3 at PPM 104.5. The chemical shifts of protons H-2′ and H-6′ at PPM 7.26 were found correlated with C-2′ and C-6′ at 127.5 PPM, while the protons (H-3′ and H5′) at 7.18 PPM were correlated with C-3′ and C-5′ at 128.9 PPM.

The HMBC data revealed some long-range correlations. One such correlation was that of singlet at 6.71 PPM (1H, s, H-3) with carbon at 163.6 PPM (C-2). Similarly, another such correlation was found between doublet protons showing signals at 7.26 PPM (1H, d, H-2′ and H-6′) and 02 quaternary carbons C-4′ at 137.6 PPM and C-2 at 163.6 PPM. The same protons (H-2′ and H-6′) also showed correlation with carbon atoms (C-6′ and C-2′) at 127.5 PPM. These data suggested that the substituents were present on C-4′. HMBC showed another correlation regarding aromatic proton on H-3′ and H-5′ at 7.18 PPM with quaternary carbon on C-1′ at PPM 127.0.

#### 3.3.4. Structure Elucidation of Compound 4

**Figure 5:**
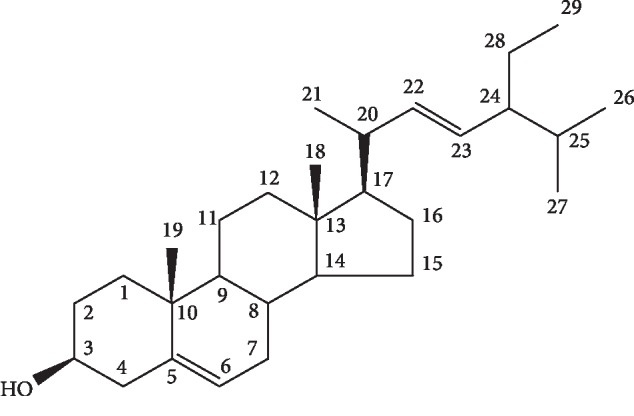
Chemical structure of *β*-sitosterol.

The purified compound 4 was acquired from chloroform-hexane fraction and analyzed by spectroscopic studies (MP 134.5°C). The electron mass spectra showed peaks at *m*/*z* 399, 397, 381, 329, and 303 conforming to the molecular formula C_29_H_50_O. The last 2 ions were accounted for sterols with unsaturation. Some more significant fragments were found at *m*/*z* 27.3 and 2.55, respectively, demonstrating the M-side chain-H_20_ loss. The MS data of *β*-sitosterol is supported by another study, where structural characterization of various phytosterols and triterpenoids isolated from black nonglutinous rice was carried out [27] Through physical and spectral data, compound 4 was recognized as *β*-sitosterol as revealed in Figure 5, which is a known compound. The ^1^H-NMR (400 MHz, CD_3_OD) and ^13^C-NMR (CD_3_OD, 100 MHz) data of compound 4 are shown in Table 5 which is supported by previously published data [28].

The present work describes the isolation of different compounds from *W. amherstiana* using chromatographic techniques and their biological evaluation. The compounds were analyzed and identified through ^1^H-NMR; various compounds which were isolated and identified were *β*-sitosterol, rutin, and quercetin. These are the compounds with known structure and reported pharmacological activities. A new compound isolated was identified as 6,7,4′-trimethyl ﬂavone (TMF). No information is available regarding its structure and biological activities. In the current research work, its structure elucidation, identification, and biological activities such as antibacterial, antifungal, cytotoxic, and antioxidant were evaluated.

### 3.4. Antibacterial Activity

Antibacterial activity was evaluated against various standard bacterial strains as shown in Table 6. TMF was tested in all concentrations as mentioned in Materials and Methods. It is necessary to mention that only the optimized concentrations (concentration at which maximum inhibitory zone is observed) were considered. TMF showed activity against *S. pneumoniae* NCTC 7466 at 300 *μ*g/ml with the inhibitory zone of 16 ± 0.29 mm in comparison with 22 ± 0.55 mm by cefazolin. Against *B. subtilis* and *S. typhi*, inhibitory zones observed were 14 ± 0.35 mm and 14 ± 0.51 mm, respectively, at 400 *μ*g/ml concentration.

**Figure 6:**
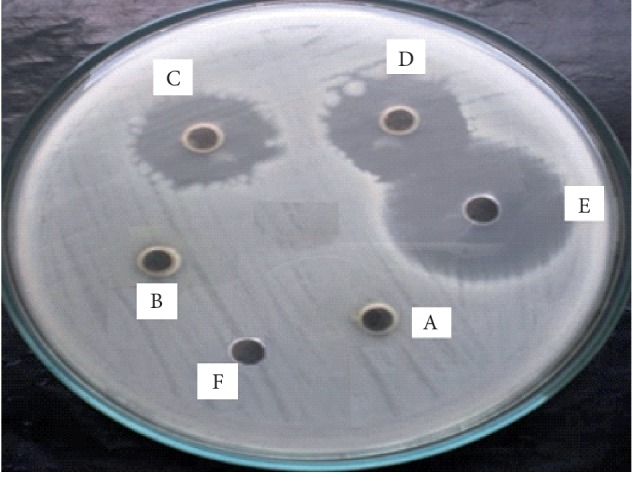
Antibacterial activity of TMF with zones of inhibition against *Staphylococcus aureus* NCTC 6571. (A) 50 *μ*g/ml, (B) 100 *μ*g/ml, (C) 200 *μ*g/ml, (D) 300 *μ*g/ml, (E) cefazolin, and (F) ethanol.

The highest activity observed by this compound (at 200 *μ*g/ml) was against *S. aureus* NCTC 6571 and the inhibitory zone observed was 18 ± 0.46 mm with 85.7% inhibition as revealed by Figure 6. The test compound was found least effective against *S. flexneri* ATCC 12022 with 52.5% inhibition. Against *P. aeruginosa* ATCC 10145, 64% inhibition was observed at 600 *μ*g/ml.

It is clearly evident from Figure 6 that at 200 and 300 *μ*g/ml, the zones of inhibition were the same. Increase in the concentration did not affect the size of the zone of inhibition. Therefore, 200 *μ*g/ml was considered as most effective concentration against *S*. *aureus*. The same procedure was adopted in case of other bacterial strains as well.

As mentioned earlier that TMF is a new compound, no literature has been reported for its antibacterial activity. From the results of the current study, its activities can be compared to the compounds extracted from other plants of the same family. A plant known as *Antirrhinum majus* of the same family had shown antibacterial and antifungal activities because of the various compounds present in it, namely, lypipnin, lutein, chrysoeriol, kaempferol-3-gluscoside, and kaempferol-3,7-diglucoside. All of these compounds were flavonoids in nature [29]. Similarly, another compound known as aurone, isolated from this plant, has demonstrated antibacterial and antifungal activities. This was a heterocyclic compound which was flavone just like TMF. In a study, various analogues of aurone were assessed for their activities against various bacteria such as *S. aureus*, *B. subtilis*, *C. tetani*, *E. coli*, *P. aeruginosa*, and *V. cholerae* and fungal strains such as *C. albicans* and *A. clavatus*. Though all these analogues were found effective against all these strains, the highest activity was found against *S. aureus* and fungal strains against *C. albicans* and *A. clavatus* [30].

### 3.5. Antifungal Assay

**Figure 7:**
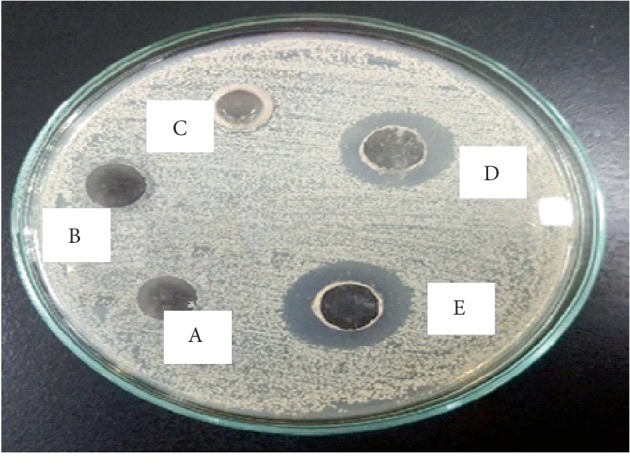
Antifungal activity of TMF with zones of inhibition against *Candida albicans* NCTC 6571, (A) ethanol, (B) 50 *μ*g/ml, (C) 100 *μ*g/ml, (D) 200 *μ*g/ml, and (E) cefazolin.

The antifungal activity of TMF against all the fungal strains is shown in Table 7. The same methodology was adopted as mentioned in antibacterial assay to find the effective concentration. The compound was found very active against *T. longifusis* clinical isolate at 400 *μ*g/ml with 90% inhibition. It was found active against *A. flavus* ATCC 32611 with 87% inhibition at 300 *μ*g/ml. It was effective against *M. canis* ATCC 11622 at 500 *μ*g/ml with 83% inhibition. Against *T. longifusis*, 90% inhibition was observed at 400 *μ*g/ml. It showed 84% inhibition against *F. solani* ATCC 11712 at 600 *μ*g/ml. At the lowest concentration, i.e., 200 *μ*g/ml, the test compound was found most effective against *C. albicans* ATCC 2091 (91.6% inhibition) among all the fungal strains as shown in Figure 7. Despite the 90% inhibition observed against *T. longifusis* at 400 *μ*g/ml, 91.6% inhibition against *C. albicans* was observed at 200 *μ*g/ml which was less than effective concentration for *T. longifusis*.

The result in Figure 7 revealed that the maximum zone of inhibition was seen at 200 *μ*g/ml by TMF. This concentration was the most effective one, as with the increase in the concentration of TMF, no rise in the inhibitory zone was observed. Here, we have considered only those concentrations, at which maximum inhibition has appeared.

The present antifungal data of TMF can be weighed against the antifungal data of other plants of the same family like *Scoparia dulcis*. In one study, ethanolic extract of this plant had shown activity against various fungal strains such as *A. niger*, *S. cerevisiae*, and *C. albicans*. The phytochemical screening of the plant ethanolic extract indicated the presence of various chemical constituents such as alkaloids, tannins, terpenes, and flavonoids that may be the reason for its antifungal activity [31]. Similarly, aqueous and methanolic extracts of *Picrorhiza kurroa*, another plant of the same family, were found active against *C. albicans* and *A. niger* [32]. The phytochemical analysis revealed that the iridoid glycosides were present in it. These glycosides were reported to have antifungal activities [33]. Another plant belonging to the same family known as *Limnophila indica* was also assessed for its antifungal activity against *Alternaria solani*, and *C. albicans* was found effective against this pathogenic fungi. These activities were attributed to the two flavones named 5,7-dihydroxy-4′,6,8-trimethoxyflavone and 5,6-dihydroxy-4′,7,8-trimethoxyflavone [34].

### 3.6. Minimum Inhibitory Concentration (MIC)

MIC of TMF was determined for all the bacterial and fungal strains under study as presented in Table 8. The results show that the MIC of this compound against *S. pneumoniae* NCTC 7466, *B. subtilis* NCTC 8236, and *S. typhi* ATCC 6539 was 256 *μ*g/ml, while its MIC against *S. flexneri* ATCC 12022 and *P. aeruginosa* ATCC 10145 was 512 *μ*g/ml. The lowest MIC was observed in case of *S. aureus* NCTC 6571, i.e.,128 *μ*g/ml proving this compound had maximum potential against the said strain.

The results revealed that the MIC of TMF against *T. longifusis* and *A. flavus* ATCC32611 was 256 *μ*g/ml. Its MIC against *M. canis* ATCC11622 and *F. solani* ATCC11712 was 512 *μ*g/ml, which was higher than all other strains. The lowest MIC (128 *μ*g/ml) was observed for *C. albican*s ATCC2091, which indicated that TMF is more active against these fungal strains than others.

TMF's MIC can be compared to other flavonoids that are extracted from the plants of the same family. A compound known as aurone which is a flavonoid was isolated from *Antirrhinum majus*. In one study, its 26 analogues were synthesized; their activities were tested against various bacterial strains such as *S. aureus* (MTCC96), *B. subtilis* (MTCC441), *C. tetani* (MTCC 449), *E. coli* (MTCC443), *P. aeruginosa* (MTCC1688), and *V. cholerae* (MTCC3906) and their MICs were determined. MICs of most of the analogues are found to lie between 250 and 500 *μ*g/ml against *S. aureus* (MTCC96), while a few of them have MIC even less than 100 *μ*g/ml proving their higher efficacy against the said strain. Out of 26 analogues, 11 analogues had MIC either 1000 *μ*g/ml or more than 1000 *μ*g/ml *C. albicans*, while one of the analogues had MIC of 100 *μ*g/ml against *C. albicans* [30].

### 3.7. Cytotoxic Activity

The brine shrimp lethality assay was used primarily to evaluate the cytotoxic activity of the newly isolated compound. In this regard, LD_50_ of TMF was calculated to be 127.01 *μ*g/ml compared to that of etoposide (8.997 *μ*g/ml). It is evident from the results summarized in Table 9 that the greater the concentration of the compound, lesser the number of survived brine shrimp larvae. At 1000 *μ*g/ml, the compound was found most cytotoxic as it killed 27 shrimps showing 90% lethality. The mortality was even greater than the standard drug, etoposide, where the mortality was 80% at the same concentration. Similarly, at 750 *μ*g/ml, the mortality was 80% which was comparatively greater than etoposide, i.e.,76.7%. About 21 shrimps were killed by TMF at 500 *μ*g/ml (mortality 70%) which was less in comparison to standard drug (73.3%). The same trend was observed with the rest of concentrations of the compound in comparison with the standard drug. However, negative control did not show any activity.

Regarding this new compound (TMF), the literature reveals no cytotoxic activity. LD_50_ of TMF came out to be 127.014 *μ*g/ml which was very less than that with the standard drug, revealing its moderate cytotoxic activity [35]. In another study, the ethanolic extract of *Scoparia dulcis* belonging to the same family was assessed for its cytotoxic activity through brine shrimps lethality assay. LD_50_ of the extract came out to be 40.39 *μ*g/ml [31], making it strongly cytotoxic [35]. The cytotoxicity was found associated with a flavone known as 5,7-dihydroxy-3′,4′,6,8-tetra-methoxyflavone present in it [36]. In another study, methanolic extracts of 13 *Verbascum* species (Scrophulariaceae) were evaluated for their cytotoxic potential through brine shrimp lethality assay. The results revealed that *V. chlorophyllum* flowers and leaves, flowers of *V. cilicicum*, *V. lasianthum*, *V. mucronatum*, *V. pycnostachyum*, and *V. splendidum* demonstrated significant cytotoxic activities [37]. It was clearly evident in the study that the flowers of the plants were more cytotoxic than the leaves; this may be due to the higher percentage of biological compounds, mostly secondary metabolites in flowers than the leaves. The phytochemical screening has indicated that the presence of saponins and glycosides in the flowers may be responsible for the cytotoxic activity among the said species [37].

### 3.8. Antioxidant Activity

**Figure 8:**
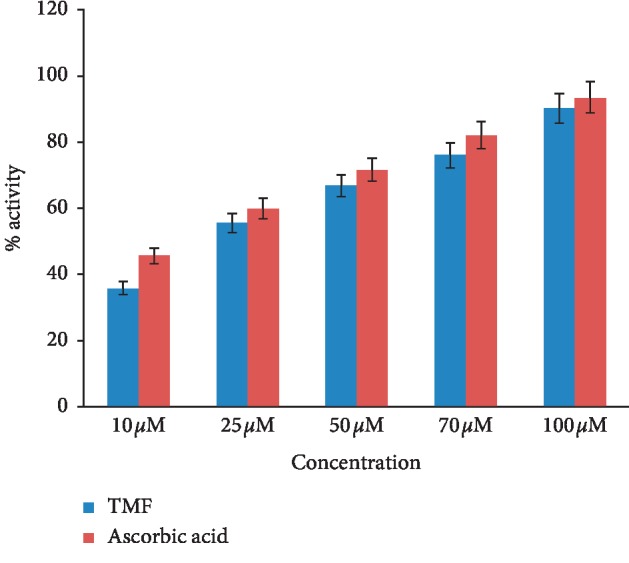
Antioxidant activity of TMF.

The antioxidant activity of the compound was determined through DPPH radical scavenging activity (RSA) as shown in Figure 8. The results revealed that RSA of TMF increases with the increase in its concentration. Its antioxidant activity was compared with ascorbic acid. The results clearly proved that the compound under study possessed antioxidant activity.

Like all other activities, no literature is available for antioxidant activity of TMF isolated from *W. amherstiana*. However, its antioxidant activity can be compared to the compounds isolated from other plants of the same family such as *Linaria vulgaris*. The antioxidant activity of *Linaria vulgaris* was assessed through 2,2-diphenyl-1-picrylhydrazyl (DPPH) radical, superoxide radical, hydroxyl radical, hypochlorous acid, and nitric oxide radical scavenging activity. The results revealed that this plant possessed remarkable scavenging capacity for superoxide, very intense scavenger of nitric oxide, and 2,2-diphenyl-1-picrylhydrazyl (DPPH) [38]. This activity may be related to various compounds isolated from this plant such as phenolic compounds and organic acids which have high antioxidant activity. Similarly, *Lindernia anagallis*, another genus of the same family, contains essential oil having thymol that has the remarkable antioxidant potential [39, 40]. *Lindernia ciliata*, another plant of the same family, contains beta-sitosterol, stigma-sterol, and lup-20(29)-en-3-beta-ol that were found in the petroleum ether fraction of the whole plant [41]. Its strong antioxidant and hepatoprotective effects were found associated with flavonoids and phenolic compounds present in it [42].

## 4. Conclusion

This study concluded that 6,7,4′-trimethyl flavone (TMF) isolated from *W. amherstiana* is a new compound as no data is reported about this compound. Our study has proved that this compound has strong antibacterial activity against *S. aureus* (85.7% inhibition), impressive antifungal activity against *C. albicans* (91.6% inhibition), and remarkable antioxidant and moderate cytotoxic activities.

## Figures and Tables

**Table 1:** Preliminary phytochemical screening.

Flavonoid test	Alkaloid test	Steroid test	Saponin test	Triterpenoid test
+ve	−ve	+ve	+ve	+ve

**Table 2:** ^13^C-NMR and ^1^H-NMR data of rutin.

No.	^13^C-NMR (PPM)	^1^H-NMR (PPM)
1		
2	156.4	
3	135.1	
4	178.2	
5	161.8	12.56 (1H, s)
6	98.3	6.18 (1H, d), *J* = 1.8 Hz
7	162.8	10.81 (1H, s)
8	94	6.41 (1H, d), *J* = 1.8 Hz
9	158.8	
10	104.5	
1′	122.8	
2′	115.3	7.54 (1H, d), *J* = 2.1 Hz
3′	145.9	9.67 (1H, s)
4′	146.5	9.18 (1H, s)
5′	157.2	6.85 (l H, d), *J* = 8.8 Hz
6′	121.8	7.56 (1H, dd), *J* = 9.0 Hz, 2.1 Hz
1″	109.8	5.30 (1H, m)
2″	75.4	3.71 (1H, m)
3″	76	3.43 (1H, m)
4″	72.9	1.84 (2H, m)
5″	75.8	3.92 (1H, m)
6″	69.5	3.38 (2H, d), *J* = 2.6 Hz
1‴	112	5.19 (1H, d), *J* = 1.6 Hz
2‴	73.8	3.73 (1H, m)
3‴	72.4	3.49 (1H, m)
4‴	73.7	3.44 (1H, m)
5‴	74.2	3.85 (1H, d), *J* = 2.4 Hz
6‴	60.6	3.85 (3H, d), *J* = 6.8 Hz

*J* is the coupling constant and is measured in Hz.

**Table 3:** ^13^C-NMR and ^1^H-NMR data of quercetin.

No.	^13^C-NMR (PPM)	^1^H-NMR (PPM)
1		
2	147.4	
3	136.2	(1H, s)
4	176.4	
5	161.2	(1H, s)
6	98.7	6.07 (1H, d), *J* = l.8 Hz
7	164.4	7.7 (1H, s)
8	93.8	6.28 (1H, d), *J* = 1.8 Hz
9	157.6	
10	98.7	
1′	122.5	
2′	115.6	7.62 (1H, d), *J* = 8.51 Hz
3′	145.5	
4′	148.2	
5′	116.1	6.78 (lH, d), *J* = 8.4 Hz
6′	115.7	7.53 (1H, d), *J* = 8.40 Hz

*J* is the coupling constant and is measured in Hz.

**Table 4:** ^13^C-NMR and ^1^H-NMR data of TMF.

No.	^13^C-NMR (PPM)	^1^H-NMR (PPM)
1	127	
2	163.6	
3	104.5	6.71 (1H, s)
4	182.1	
5	162.1	12 (1H(OH), s)
6	117.2	
7	144.7	
8	106.5	6.60 (1H, s)
9	155.5	
10	109.8	
11	11	1.2 (3H, s)
12	19.4	1.51 (3H, s)
1′	127	
2′	127.5	7.26 (1H, d), *J* = 8.51 Hz
3′	128.9	7.18 (1H, s)
4′	137.6	
5′	128.9	7.18 (1H, d) (8.81 Hz)
6′	127.5	7.26 (1H, s)
7′	21.3	1.51 (3H, s)

*J* is the coupling constant and is measured in Hz.

**Table 5:** ^13^C-NMR and ^1^H-NMR data of *β*-sitosterol.

No.	^13^C-NMR (PPM)	^1^H-NMR (PPM)
1	37.8	
2	40.7	
3	72.9	3.5 (1H, m)
4	43	
5	140.7	35.7 (1H, t)
6	122	
7	32.8	
8	32.1	
9	51.2	
10	37.1	
11	21.7	
12	41.1	
13	43	
14	57.6	
15	26.9	
16	29	
17	56.9	
18	36.8	
19	18.9	0.96 (3H, d), *J* = 6.8
20	34.7	
21	24.9	
22	46.6	
23	23.7	
24	12.6	0.87 (3H, t), *J* = 7.8 Hz
25	30	
26	20.4	0.80 (3H, d), *J* = 5.9 Hz
27	19	0.80 (3H, d), *J* = 6.8 Hz
28	18.9	0.69 (3H, s)
29	12.6	0.96 (3H, s)

*J* is the coupling constant and is measured in Hz.

**Table 6:** Zone of inhibition of TMF and antibiotics used.

Bacterial strains	Zone of inhibition (mm)		
	TMF	Standard drug	% Inhibition
*Streptococcus pneumoniae* NCTC 7466	16 ± 0.29	22^*∗*^ ± 0.55	72.2
*Bacillus subtilis* NCTC 8236	14 ± 0.35	18^*∗*^ ± 0.62	77.7
*Staphylococcus aureus* NCTC 6571	18 ± 0.46	21^*∗*^ ± 0.37	85.7
*Shigella flexneri* ATCC 12022	10.5 ± 0.20	20^*∗∗*^ ± 0.63	52.5
*Pseudomonas aeruginosa* ATCC 10145	16 ± 0.42	25^*∗∗*^ ± 0.76	64
*Salmonella typhi* ATCC 6539	14 ± 0.51	26^*∗∗*^ ± 0.26	54

^*∗*^Cefazolin, ^*∗∗*^kanamycin, TMF = 6,7,4′-trimethyl ﬂavone. The experimental values are expressed as means in triplicate, *n* = 3 with ± standard error of mean (±SEM).

**Table 7:** Zones of inhibition of TMF and standard drug used.

Fungal strains	Zone of inhibition (mm)		
	TMF	Standard drug	% Inhibition
*Trichophyton longifusis* clinical isolate	15 ± 0.71	16.5^*∗*^ ± 0.65	90
*Aspergillus flavus* ATCC 32611	18 ± 0.33	20.5^*∗∗*^ ± 0.58	87
*Microsporum canis* ATCC 11622	20 ± 0.49	24^*∗*^ ± 0.45	83
*Fusarium solani* ATCC 11712	16 ± 0.65	19^*∗*^ ± 0.76	84
*Candida glabrata* ATCC 60406	17.5 ± 0.58	21^*∗*^ ± 0.33	83.3
*Candida albicans* ATCC 2091	16.5 ± 0.34	18^*∗*^ ± 0.37	91.6

^*∗*^Miconazole, ^*∗∗*^amphotericin-A, and TMF = 6,7,4′-trimethyl ﬂavone. The experimental values are expressed as means in triplicate, *n* = 3 with ±standard error mean (±SEM).

**Table 8:** Minimum inhibitory concentration (MIC) assay of TMF.

	MIC (*μ*g/ml) TMF
*Bacterial strains*	
*Streptococcus pneumoniae* NCTC 7466	256 ± 0.82
*Bacillus subtilis* NCTC 8236	256 ± 0.78
*Staphylococcus aureus* NCTC 6571	128 ± 0.94
*Shigella flexneri* ATCC 12022	512 ± 0.69
*Pseudomonas aeruginosa* ATCC 10145	512 ± 1.02
*Salmonella typhi* ATCC 6539	256 ± 0.89

*Fungal strains*	
*Trichophyton longifusis* clinical isolate	256 ± 0.79
*Aspergillus flavus* ATCC 32611	256 ± 0.91
*Microsporum canis* ATCC 11622	512 ± 1.12
*Fusarium solani* ATCC 11712	512 ± 0.98
*Candida albicans* ATCC 2091	128 ± 0.63
*Candida glabrata* ATCC 60406	256 ± 0.82

TMF = 6,7,4′-trimethyl ﬂavone. The experimental values are expressed as means in triplicate, *n* = 3 with ±standard error of mean (±SEM).

**Table 9:** Brine shrimp lethality assay (cytotoxic activity of TMF).

Material	Dose (*μ*g/ml/ml)	No. of shrimps	No. of survivors	Mortality %	LD_50_ (*μ*g/ml)
*6,7,4′-trimethyl ﬂavone*	1000	30	3	90	127.014
	750	30	6	80	
	500	30	9	70	
	100	30	15	50	
	50	30	21	30	

*Positive control etoposide*	1000	30	6	80	8.997
	750	30	8	76.7	
	500	30	8	73.3	
	100	30	10	66.7	
	50	30	12	60	

*Negative control sea water*	1000	30	30	0	—
	750	30	30	0	
	500	30	30	0	
	100	30	30	0	
	50	30	30	0	

## Data Availability

All the research data used to support the findings of this study are included within the article.
